# The changes in multi-scale structure and properties of wheat starch after interaction with oligosaccharides of different polymerization degrees under different freeze-thaw cycles

**DOI:** 10.1016/j.fochx.2025.102482

**Published:** 2025-04-24

**Authors:** Juanjuan Guo, Zengming Huang, Peilin Chen, Xiantong Wu, Xu Lu

**Affiliations:** a College of Oceanology and Food Sciences, Quanzhou Normal University, Quanzhou, Fujian 362000, China; b Huitouke Food Group Co., Ltd, Quanzhou, Fujian 362000, China; c College of Food Science, Fujian Agriculture and Forestry University, 15 Shangxiadian Road, 350002 Fuzhou, China; d Fujian Province Key Laboratory for the Development of Bioactive Material from Marine Algae, Quanzhou 362000, China

**Keywords:** Wheat starch, Freeze-thaw cycle, Oligosaccharides, Retrogradation

## Abstract

This study investigated the effects of three oligosaccharides, stachyose (STA), raffinose (RAF), and sucrose (SUC), all at 16 % *w*/w, on the physicochemical properties of wheat starch subjected to multiple freeze-thaw (FT) cycles. The findings revealed that all three oligosaccharides reduced starch particle size and mitigated the degradation of endogenous nutrients, such as proteins and lipids, typically induced by repeated FT treatments. Additionally, the oligosaccharides decreased the content of amylose, crystallinity and short-range molecular order of starch, as well as the gelatinization enthalpy (*ΔH*) and rapidly digestible starch. They also suppressed granule swelling, leading to reductions in peak, breakdown, and setback viscosities, thereby enhancing the shear resistance of the starch paste. Among the tested oligosaccharides, SUC was the most effective in preserving starch integrity and nutrient content, whereas STA had a greater capacity to reduce crystallinity and stabilize viscosity parameters. These results provide a theoretical foundation for optimizing the use of oligosaccharides in frozen wheat starch-based food products.

## Highlights

• Freeze-thaw promotes the recrystallization of the starch crystal region.
• Oligosaccharides can reduce starch retrogradation.
• STA could more effectively inhibit the retrogradation of freeze-thaw wheat starch.
• The antifreeze effect of oligosaccharides is related to its structure.

## 1 Introduction

Freezing is a crucial preservation method, as frozen dough typically requires only thawing and baking before being served to consumers. However, during transportation, the dough is frequently exposed to fluctuations between freezing and ambient temperatures, leading to the recrystallization of ice crystals. This process weakens the gluten structure, reduces yeast activity, and ultimately diminishes the sensory properties and overall quality of the dough product. Research has indicated that the hardness of rice starch gels subjected to repeated freeze–thaw cycles increases (Charoenrein et al., 2010). Similarly, Jankowski observed that the gel network of potato starch became harder and less sticky after freezing (Jankowski, 1992). As wheat starch constitutes over 70 % of wheat flour (Tao, Wang, Wu, et al., 2016), its contribution to food quality is primarily determined by its physical and chemical properties in the presence of water. These properties, such as gelatinization and rheological characteristics, affect the viscosity, texture, consistency, nutrition, taste, and shelf life of starch-based products. The characteristics of dough, including hardness, elasticity, chewability, and overall texture, are influenced by the physicochemical properties of wheat starch, such as viscosity and swelling power. For instance, certain physicochemical properties of wheat starch affect moisture absorption in the dough, which in turn affects the hardness of baked products, ultimately influencing the quality of flour-based goods. However, the recrystallization of ice crystals in frozen dough during prolonged storage disrupts the structure of starch granules (H. Wang et al., 2021), damaging the crystallization areas within the starch and accelerating retrogradation (Szymońska & Wodnicka, 2004). Additionally, the freezing process alters the movement of moisture within the starch granules, compressing the starch structure and disrupting its nutrient components such as proteins and lipids. This leads to the leaching of endogenous nutrients and accelerates retrogradation in frozen starch products (Tao et al., 2015). The formation of ice crystals during freezing damages starch granules and leaches amylose. Upon thawing, amylose and amylopectin chains tend to re-associate, leading to recrystallization. This process diminishes yeast activity, weakens the gluten network, and adversely affects the sensory attributes of the product, making it difficult for the dough to regain its original characteristics (Tao, Wang, Wu, et al., 2016).

It has been established that the properties of frozen starch are influenced not only by the molecular structure of the starch (i.e., the amylose-to-amylopectin ratio) and the concentration of starch solutions, but also by environmental factors such as temperature and non-starch components including salt, sweeteners, lipids and hydrocolloids. Among these, sweeteners play a particularly significant role. The presence of sweeteners can alter both the gelatinization and retrogradation behaviors of starch. In certain baked goods (e.g., cakes, breads, rolls, and muffins), starch gelatinization or swelling contributes to the desired structure and texture (Godefroidt et al., 2019). Conversely, in products such as sugar cookies and shortbread, excessive gelatinization or swelling of starch may lead to undesirable quality traits. Sweeteners modify the thermal properties of starch, generally increasing the gelation and gelatinization temperatures, delaying starch granule swelling, and altering the extent of swelling. However, excessive intakes of sweeteners, salt, and lipids pose potential health risks to consumers. Functional oligosaccharides, which are highly water-soluble and possess low sweetness, are promising alternatives to traditional sweeteners, particularly for individuals with diabetes, cardiovascular conditions, or thrombosis. Research has demonstrated that sucrose (SUC) increases the gelatinization temperature of wheat starch more effectively than many other sweeteners (Allan et al., 2018), thus reducing the overall sugar content in formulations while still achieving a higher gelatinization temperature that limits starch gelatinization. Furthermore, studies have shown that several indigestible oligosaccharides, at comparable concentrations, can increase the gelatinization temperature as much as or more than sucrose without altering the enthalpy change (*ΔH*) (Woodbury & Mauer, 2022). The impact of oligosaccharides on starch regeneration is concentration-dependent and varies according to the type of oligosaccharide used (Allan and Mauer, 2022). Given these effects, exploring the influence of different oligosaccharides on wheat starch properties holds significant potential. Certain prebiotic sources, such as indigestible carbohydrates, particularly oligosaccharides like raffinose (RAF, formed by *α*-D-galactosyranose-(1 → 6)-*α*-d-glucopyranose-(1 → 2)-*β*-d-fructose-furanosinoside)and stachyose (STA, formed from *α*-D-galactopyranoside-(1 → 6)-*α*-d-galactopyranose-(1 → 6)-*α*-D-glucopyranoscopyranos-(1 → 2)-*β*-D-fructofuranoside), fit the criteria for prebiotic classification and can confer health benefits to the host. These low-molecular-weight oligosaccharides penetrate starch granules at ambient temperatures, reducing amylose leaching and starch swelling, thereby enhancing its stability (D.-N. Zhou et al., 2017). Investigating the interactions between functional oligosaccharides and starch under frozen conditions could provide valuable insights into the development of frozen flour-based products with prebiotic functionalities.

Recent studies have examined the effects of various oligosaccharides on the properties of wheat starch. Oligosaccharides form stable hydrogen bonds with starch chains in the non-crystalline regions of starch granules at ambient temperature. This interaction reduces the density of hydrogen-bond sites and plasticization capacity in the solvent (Woodbury, Grush, Allan and Mauer, 2022). Oligosaccharides, such as trehalose and carrageenan, can delay the increase in the relative content of *β*-fold and decrease the relative content of *α*-helix in starch. Additionally, they reduce the size of pores in gluten protein, leading to a more uniform structure, thereby enhancing the integrity and continuity of the network structure. ASKG (a specific oligosaccharide) has been shown to significantly limit the swelling of starch granules, improve the relative crystallinity of starch molecules, and reduce the swelling power of gels and gelatinization viscosity (Shan et al., 2023; Shen et al., 2025). Despite these advances, research on the mechanisms underlying the interactions between oligosaccharides with a single degree of polymerization and starch during different freeze-thaw cycles is limited. Most studies investigating the effects of oligosaccharides on wheat starch regeneration have focused on low additive concentrations (<5 %*w*/w), particularly in terms of bread functionality (Akers, & Hoseney., R. C., 1994; Durán et al., 2001). In contrast, research on higher concentrations, which are typically found in sweet baked goods, remains sparse. Furthermore, much of the research has isolated starch for analysis, rather than considering starch within the context of frozen dough systems. The formation of dough involves the creation of a three-dimensional gluten network, with starch granules and water embedded within it, creating a complex system (Jekle et al., 2016). Additionally, starch may interact with other components, such as proteins and lipids, within the dough matrix. In this study, we selected three oligosaccharides, sucrose (SUC), raffinose (RAF), and stachyose (STA), which differ slightly in their degrees of polymerization. These oligosaccharides were selected to facilitate the identification of structural differences between them. Both RAF and STA are recognized as prebiotic oligosaccharides with probiotic properties. This study investigated the effects of these functional oligosaccharides (STA, RAF, and SUC) at an additive concentration of 16 % (*w*/w) on the composition, structure, gelatinization, and rheological properties of wheat starch granules after multiple freeze-thaw cycles.

## 2 Materials and methods

### 2.1 Materials

Wheat starch (89.3 % dry matter) was procured from Shanghai Lvyuan Starch Co., Ltd. (Shanghai, China). SUC, consisting of *α*-d-glucopyranosyl-(1 → 2)-*β*-D-fructofuranoside, RAF, and STA, all with a purity greater than 98 %, were obtained from Sigma-Aldrich Chemical Co. (St. Louis, MO, USA). All other chemicals and reagents were obtained from Sinopharm Chemical Reagent Co., Ltd. (Shanghai, China) and were of analytical grade unless otherwise specified.

### 2.2 Methods

#### 2.2.1 Preparation of frozen starch

A mixture of 8 g each of SUC, RAF, and STA was combined with 50 g of wheat starch (Luyuan Starch Co., Ltd., Shanghai, China) and 75 mL of deionized water. The mixture was stirred for 30 min, followed by a 3 h rest period. The resulting starch-oligosaccharide solution was then placed in a freezer at −20 °C for 24 h, followed by thawing at 25 °C for 12 h, completing one freeze-thaw cycle. The freeze-thaw process was repeated for 3, 7 and 11 cycles, respectively. Subsequently, the solution was stored at −20 °C for 7 days and thawed at room temperature. The solution was centrifuged at 5000*g* for 10 min, and the starch pellet was collected to remove the free oligosaccharides from the supernatant. The pellet was magnetically stirred in water for 15 min and centrifuged; this procedure was repeated three times. The final starch precipitate was freeze-dried (FD4C80, Fuyikang Co., Ltd., Beijing, China), crushed, and sieved through a 100-mesh screen (Su et al., 2020).

#### 2.2.2 Determination of damaged starch content (DS)

The damaged starch content (%) was determined according to the AACC 76–31.01 method using a Starch Damage Assay Kit (K-SDAM) purchased from Megazyme International Ireland Ltd. (Co. Wicklow, Ireland). All samples were analyzed in triplicates.

#### 2.2.3 Determination of amylose starch content

The amylose content was determined using a dual-wavelength iodine binding technique. 10 mg of starch was weighed and dissolved in 0.1 mL ethanol. 1 mL of sodium hydroxide (1 mol/L) was added and stirred for about 1 h to ensure that the starch slurry was completely clear with no visible clumps. The solution was then diluted to 10 mL of distilled water, 0.2 mL of this dilution was further diluted with 5 mL of distilled water in another 10 mL volumetric flask. With phenolphthalein as an indicator, the solution was titrated with HCl (0.1 mol/L) to neutrality. Then 0.2 mL of 0.2 % iodine solution was added and diluted the final solution to 10 mL by adding distilled water, which left at room temperature for 30 min to develop the color. Finally, absorbance values at 620 nm and 510 nm were determined using a UV–Vis spectrophotometer. The absorbance is linear with the percentage of amylose. The amylose content is calculated by eq. (1):

(1) $$\text{Amylose}\left(\%\right)=\frac{\Delta {А}_{ABS}–0.01526}{0.0028}$$

where *ΔА*_ABS_ represents the difference between absorbance values at 620 nm and 510 nm (ABS 620-ABS 510).

#### 2.2.4 True densities of frozen starch granules

The true density (*T*_*d*_) of the frozen starch granules was measured using a specific-gravity bottle (AccPyc II 1340, Micromeritics Co., GA, USA). The starch was ground into a fine powder, sieved through a 100 mesh screen, and dried for 24 h. The dried starch was then compressed into a 50 mL cylindrical container, and its volume was measured using the helium displacement method. The moisture content of the starch samples was approximately 10 %.

#### 2.2.5 Bulk densities and porosity of frozen starch granules

The bulk density was determined by measuring the volume of 50 g of starch solids in a 100 mL graduated cylinder. Bulk density was calculated as the ratio of weight to volume. Porosity (*P*_*f*_) was calculated using the following formula:

(2) $$P_{f}=\left(1-\frac{B_{d}}{T_{d}}\right)\times 100$$

where *B*_*d*_ is the bulk density, and *T*_*d*_ is the true density.

#### 2.2.6 Particle size distribution (PSD) of frozen starch granules

The particle size distribution (PSD) of the frozen starch granules was analyzed using a laser diffraction particle size analyzer (MasterSizer 3000; Malvern Co., Ltd., Worcestershire, UK). The starch sample was dispersed in deionized water and agitated at 2000 rpm to ensure homogeneity (absorption index of 0.001 and a refractive index of 1.330). The average particle diameter was calculated as D[4,3], which is more sensitive to larger particles.

(3) $$D\left[43\right]=\frac{\sum_{i}n_{i}d_{i}^{4}}{\sum_{i}n_{i}d_{i}^{3}}$$

#### 2.2.7 Scanning electron microscopy (SEM) of frozen starch granules

Starch granule powders were applied to adhesive tape and gold-coated using an argon plasma metallizer (sputter coater PELCO 91000). The samples were then examined using a scanning electron microscope (Nova Nano SEM 230, SRO Co., Ltd., Brno, Czech Republic) at an acceleration voltage of 10 kV (FuZhen et al., 2023).

#### 2.2.8 X-ray diffraction (XRD) for frozen starch granules

The crystalline structure of the starch samples (with consistent moisture content) was analyzed using an X-ray diffractometer (D8, Bruker, USA). Data were collected within the range of 2θ (5–40°). The relative crystallinity (*Rc*, %) was calculated using the Jade 7.0 software, following the formula:

(4) $$R_{c}=\frac{\sum_{i=1}^{n}A_{ci}}{A_{t}}=\frac{100\times A_{m}}{A_{m}+A_{s}+A_{a}}$$

*A*_*ci*_ is the area with index *i* for each crystalline peak, and *A*_*t*_ is the total area of the diffraction pattern. *A*_*m*_ is the area of the microcrystalline region, *A*_*s*_ is the area of the subcrystalline region, and *A*_*a*_ is the area of the noncrystalline region. The crystallinity, sub-crystalline region, and non-crystalline region were determined by integrating and separating the peaks corresponding to the crystalline and non-crystalline regions (Ribotta et al., 2004).

#### 2.2.9 Fourier transform infrared spectroscopy (FTIR) for frozen starch granules

The molecular double helix and short-range ordered structures of the wheat starch were analyzed using a VERTEX 70v spectrometer (Bruker, Germany). The samples were prepared using the KBr method to ensure uniform moisture content. Spectral analysis was performed using OMNIC software (Ver. 8.0, Nicolet, Wisconsin, USA) with Gaussian deconvolution to calculate the intensity ratio of the peaks at 995/1022 cm^−1^ and 1047/1022 cm^−1,^ respectively.

#### 2.2.10 Raman spectroscopy of frozen starch granules

Raman spectroscopy was conducted following the method described by Wang (H. Wang et al., 2020) with slight modifications. A small sample was placed on a glass slide, compressed, and scanned across the entire wavelength range. Spectra were recorded in the range of 3200–100 cm^−1^, with an excitation wavelength of 785 nm. The spectral data were fitted and analyzed, and the peak width at 480 cm^−1^ was determined.

#### 2.2.11 Low-field nuclear magnetic resonance (LF-NMR) for frozen starch granules

The relaxation times and water content in different regions of the wheat starch were determined using an NMR analyzer (Meso23060H, Niumag Co., Ltd., Suzhou, China). A starch suspension (3 g starch powder with 3 mL deionized water) was equilibrated for 2 h before measurement. The transverse relaxation time (*T*_*2*_) was measured using the Q-CPMG sequence (Yanlin et al., 2023), and the *T*_*2*_ spectrum was obtained through multicomponent inversion after the cumulative measurements.

#### 2.2.12 Differential scanning calorimetry (DSC) for frozen starch granules

The thermal and phase transition properties of wheat starch were analyzed using a DSC (204 F1 Phoenix®, Netzsch Co., Ltd., Selb, Germany). A 3 mg sample of starch was combined with 10 μL of deionized water, transferred into an aluminum pan, and sealed with a Tzero hermetic lid (Lu et al., 2025). The sample was equilibrated overnight and then heated from 30 to 100 °C at a rate of 5 °C/min under a nitrogen atmosphere.

#### 2.2.13 Viscosity measurement (RVA) for frozen starch granules

The gelatinization properties of the starch were determined using a Rapid Visco Analyzer (4500, Perten Co., Ltd., Hägersten, Sweden). A 2.5 g sample of starch was mixed with 25 mL of deionized water in an RVA aluminum canister. The slurry was heated and cooled according to the following temperature profile: (1) held at 50 °C for 1 min, (2) heated to 95 °C at 12 °C/min for 2.5 min, and (3) cooled to 50 °C at 12 °C/min for 2 min.

#### 2.2.14 Rheological property

##### 2.2.14.1 Steady rheological properties of frozen starch paste

The rheological properties were evaluated using a rheometer (MCR301; Anton Paar, Graz, Austria). After complete gelatinization via RVA treatment followed by cooling, the starch paste was placed on the measurement platform (maintained at 25 °C), and A PP50 probe was used with a scraper to remove any excess samples. The viscosity and shear stress were measured as the shear rate varied from 0 to 100 s^−1^. The resulting rheological data were fitted to the Herschel-Bulkley model using the following equation:

(5) $$\tau =\mathrm{\tau o}+K\gamma^{n}$$

where *τ* represents shear stress (Pa), *τ*_*0*_ is the characteristic value of plastic fluid (the yield stress), *K* is the consistency coefficient (Pa · Sn), *γ* is the shear rate (s^−1^), and *n* is the flow behavior index (dimensionless).

##### 2.2.14.2 Dynamic rheological properties of frozen starch paste

To prevent water evaporation, a thin layer of silicone oil was applied to the starch paste at 25 °C. The dynamic rheological properties were analyzed using a frequency-sweep program. The linear viscoelastic region of each sample was determined by strain sweeping (0.1–100 % at a frequency of 1 Hz). Subsequently, a frequency sweep was conducted within the angular frequency range of 0.1–10 Hz at a strain of 1 %, which was the most stable condition within the linear viscoelastic region. A frequency sweep was performed within the range of angular frequency (*ω*) of 100 rad/s to determine the relationship between the storage (*G'*) and loss modulus (*G"*) with angular frequency (Qingzhong et al., 2023; Shan et al., 2023).

##### 2.2.14.3 In-shear structural recovery properties of frozen starch paste

Structural recovery was assessed using a three-phase procedure: (1) Phase 1: the shear rate was maintained at 1 s^−1^ for 2 min; (2) Phase 2: the shear rate was increased to 300 s^−1^ for 1 min; and (3) Phase 3: the shear rate was reduced back to 1 s^−1^ for 3 min. The structural recovery of the paste was quantified as the ratio of the apparent viscosity measured in Phase 1 to that measured in Phase 3.

#### 2.2.15 Digestive resistance of frozen starch

The oligosaccharide–starch systems were digested in vitro using a previously described method with some modifications (Zhang, Jiang, Pan, Lv, Liu, Zhang et al., 2018). Briefly, starch was treated with *α*-amylase in vitro and the absorbance of the hydrolyzed sample was tested by DNS method combined with spectrophotometry. The absorbance values of each group were detected at 530 nm to calculate the glucose content. The specific calculation formula is as follows:

(6) $$\mathrm{RDS}\left(\%\right)=\frac{\left(G_{20}-G_{0}\right)\times 0.9}{\mathrm{TS}}\times 100\%$$

(7) $$\mathrm{SDS}\left(\%\right)=\frac{\left(G_{120}-G_{20}\right)\times 0.9}{\mathrm{TS}}\times 100\%$$

(8) $$\mathrm{RS}\left(\%\right)=\frac{\left(\mathrm{TS}-G_{20}\right)\times 0.9}{\mathrm{TS}}\times 100\%$$

where *G*_*0*_, *G*_*20*_, and *G*_*120*_ represent the glucose content produced at 0, 20 and 120 min, respectively, and *TS* represents the total amount of starch in the sample before enzymatic digestion.

#### 2.2.16 Statistical analysis

Statistical analyses were performed using the Data Processing System (DPS, Version 9.05) software (Science Press Co., Ltd., Beijing, China). Data are presented as mean values ± standard deviation and were visualized using OriginPro software (version 2017, Origin Lab Co., Ltd., USA). Significant differences were assessed using one-way analysis of variance (ANOVA), followed by Duncan's post-hoc test. All experiments were performed in triplicate.

## 3 Results and discussion

### 3.1 The particle size distribution of frozen starch granules

Wheat starch granules are classified into two types based on particle size: A-type granules (particle size ≥10 μm) and B-type granules (particle size ≤10 μm). The D[4,3] of unfrozen wheat starch granules was (16.28 ± 0.03) μm (Table 1), indicating that the starch granules used in this study were predominantly of the A-type. The irregular shape and larger particle size of these granules may exacerbate starch damage during repeated freeze-thaw cycles. In contrast, starch granules with smaller particle sizes, which possess higher levels of phosphate groups, are likely to exhibit stronger intermolecular forces, resulting in less damage during freezing and thawing. A significant increase in the D[4,3] value was observed after freeze-thawing (*p* < 0.05), suggesting that the granules swelled further owing to the formation of ice crystals during the freeze-thaw cycles. This led to an increase in particle size, which became more pronounced with the number of freeze-thaw cycles.

**Table 1:** Particle size, true density, bulk densities, porosity, endogenous ingredients, free amylose content of native wheat starch and starch-oligosaccharides at different freezing-thawing cycles.

Index		Cycles	NWS	FTS	FTS-STA	FTS-RAF	FTS-SUC
PSD	D_[4,3]_(μm)	3	16.28 ± 0.03^a^	19.53 ± 0.02^b^	18.51 ± 0.02^c^	18.24 ± 0.04^d^	17.80 ± 0.01^e^
		7		24.42 ± 0.03^b^	18.72 ± 0.03^c^	18.53 ± 0.04^d^	17.92 ± 0.03^e^
		11		28.22 ± 0.02^b^	18.94 ± 0.03^c^	18.63 ± 0.02^d^	18.51 ± 0.01^d^
True density(g/cm^3^)		3	1.471 ± 0.002^a^	1.526 ± 0.002^b^	1.474 ± 0.003^a^	1.474 ± 0.001^a^	1.484 ± 0.002^c^
		7		1.527 ± 0.003^b^	1.482 ± 0.003^c^	1.484 ± 0.001^c^	1.491 ± 0.002^d^
		11		1.531 ± 0.003^b^	1.493 ± 0.002^c^	1.494 ± 0.003^c^	1.493 ± 0.000^c^
Bulk densities(g/cm^3^)		3	0.99 ± 0.00^a^	0.81 ± 0.00^b^	0.83 ± 0.00^c^	0.83 ± 0.00^d^	0.86 ± 0.00^e^
		7		0.80 ± 0.00^b^	0.79 ± 0.00^b^	0.81 ± 0.00^c^	0.86 ± 0.00^d^
		11		0.78 ± 0.00^b^	0.79 ± 0.00^c^	0.80 ± 0.00^d^	0.83 ± 0.00^d^
Porosity(%)		3	32.32 ± 0.04^a^	47.12 ± 0.06^b^	44.86 ± 0.08^c^	43.60 ± 0.12^c^	41.74 ± 0.06^d^
		7		47.72 ± 0.06^b^	46.89 ± 0.03^c^	45.60 ± 0.06^d^	42.49 ± 0.07^e^
		11		49.08 ± 0.01^b^	47.12 ± 0.08^c^	46.64 ± 0.09^d^	44.17 ± 0.12^e^
Starch endogenous ingredients	Protein (%)	3	0.53 ± 0.023^a^	0.12 ± 0.025^b^	0.24 ± 0.004^c^	0.37 ± 0.012^d^	0.46 ± 0.015^e^
		7		0.08 ± 0.006^b^	0.23 ± 0.004^c^	0.24 ± 0.003^d^	0.46 ± 0.004^e^
		11		0.05 ± 0.006^b^	0.1 ± 0.002^c^	0.12 ± 0.002^d^	0.20 ± 0.002^e^
	Lipid (%)	3	0.21 ± 0.004^a^	0.06 ± 0.006^b^	0.06 ± 0.001^c^	0.09 ± 0.001^d^	0.10 ± 0.001^e^
		7		0.04 ± 0.012^b^	0.05 ± 0.001^c^	0.06 ± 0.001^d^	0.08 ± 0.003^e^
		11		0.03 ± 0.006^b^	0.05 ± 0.001^c^	0.05 ± 0.001^c^	0.06 ± 0.002^d^
	Damage starch (%)	3	0.08 ± 0.004^a^	0.45 ± 0.013^b^	0.21 ± 0.006^c^	0.16 ± 0.006^d^	0.12 ± 0.012^e^
		7		0.46 ± 0.008^b^	0.27 ± 0.013^c^	0.23 ± 0.025^d^	0.21 ± 0.008^e^
		11		0.49 ± 0.009^b^	0.32 ± 0.039^c^	0.29 ± 0.011^d^	0.24 ± 0.026^e^
Amylose content	Amylose (%)	3	27.57 ± 0.22^a^	0.01 ± 0.00^b^	0.05 ± 0.00^c^	0.04 ± 0.00^d^	0.75 ± 0.01^e^
		7		10.77 ± 0.22^b^	4.72 ± 0.11^c^	4.81 ± 0.17^c^	7.32 ± 0.22^d^
		11		12.37 ± 0.38^b^	4.75 ± 0.14^c^	5.64 ± 0.05^d^	7.77 ± 0.21^e^

Mean of three measurements ± standard deviation. Values for the same processing conditions and different additive groups with different letters are significantly different (*p* < 0.05). The particle size, true density, bulk densities, porosity results of NWS group were derived from all results obtained in the reference NWS group of our study (Su et al., 2020). (NWS: native wheat starch; FTS: freezing/thawing-treated wheat starch; FTS-STA: freezing/thawing-treated wheat starch with stachyose; FTS-RAF: freezing/thawing-treated wheat starch with raffinose; FTS-SUC: freezing/thawing-treated wheat starch with sucrose.)

The addition of STA, RAF, and SUC to starch significantly reduced the particle size of the frozen starch granules compared to that of FTS (freeze-thaw treated wheat starch) (*p* < 0.05). This reduction is attributed to the lower glass transition temperature (Tg) of oligosaccharides, which lowers the freezing point of the solution. Consequently, the formation of large ice crystals in free water is restricted, preventing the extrusion and aggregation of starch granules by ice crystals, thereby decreasing the particle size (van der Sman, 2016). These findings align with those of Travelst J (J., Erica, C., & J., 2022). Among the oligosaccharides, the particle size reduction effect followed the order: SUC > RAF > STA (Table 1). This may be linked to the number of hydroxyl groups present in the oligosaccharide molecules. Hydroxyl groups interact with water molecules to form hydrogen bonds, thereby enhancing the hydration capacity of oligosaccharides. STA and RAF molecules, which have more hydroxyl groups than SUC (SUC has a lower relative molecular weight), may interact more strongly with water, resulting in higher aggregation and the formation of larger ice crystals outside the starch granules. This phenomenon likely increases the swelling ability of starch, leading to a larger particle size upon STA addition. Furthermore, STA and RAF are more rigid than SUC, potentially limiting their ability to stabilize the starch structure (Best, Jackson and Naidoo, 2001; van der Sman & Mauer, 2019), which may also contribute to the larger particle size observed with these treatments. These results indicate that the addition of SUC and treatment with fewer freeze-thaw cycles can effectively reduce the extent of particle size enlargement in frozen wheat starch granules.

### 3.2 The true densities of frozen starch granules

True density refers to the mass per unit volume of starch in a completely dense state, achieved after eliminating the space between particles and the internal pores. As the number of freeze-thaw cycles increased, the true density of natural wheat starch (NWS) gradually increased (Table 1). This enhancement is attributed to the strengthening of the helical structure within the starch molecular chain, which increases its true density. The expansion of pores within the granules, along with the tightening of the molecular spirals, contributed to this increase (Noman et al., 2023)*.*

Conversely, the true density of wheat starch significantly decreased following oligosaccharide addition (*p* < 0.05) (Table 1). This reduction is likely due to the limited expansion of ice crystals, which inhibits the increase in the true density that is typically associated with the double helix effect. Among the tested oligosaccharides, the true density of starch modified with STA was the lowest (Table 1). This may be attributed to the high number of hydroxyl groups in STA, which increases its ability to interact with both starch and water. The interactions effectively stabilize the water within the granule and reduce its mobility, thereby limiting the shrinkage of the starch granules. Furthermore, STA's larger molecular weight suggests that its larger sugar molecules may bridge longer gaps between starch chains, enhancing granule stability. This, in turn, weakens the double helix structure and lowers the true density (Kitagawa et al., 2025). Therefore, oligosaccharides effectively reduced the true density of frozen wheat starch granules, with STA having the most significant effect.

### 3.3 The bulk densities and porosity of frozen starch granules

Bulk density is closely associated with the particle size and microstructure of the starch powder. A lower bulk density indicates a looser powder with greater intergranular porosity. Porosity (*Pf*) quantifies the unoccupied space between starch granules and is influenced by the shape and size of the granules (Deepika et al., 2013). Compared to NWS, the freeze-thaw treatment significantly reduced the bulk density and increased porosity (*p* < 0.05) (Table 1), suggesting that the starch powder became more loosely packed after freezing. This change is attributed to the swelling of starch granules and their internal channels due to ice crystal formation under the action of microscopic mechanical forces during the freeze-thaw process. As the ice melted, water was released from the granule pores, resulting in a more porous structure. Furthermore, the increased number of starch granules post-thawing contributed to enhanced porosity.

Compared to FTS, the addition of oligosaccharides significantly decreased starch porosity (*p* < 0.05) (Table 1). Oligosaccharides modify the microstructure by influencing ice crystal formation. During freezing in a sugar solution, the sugar concentration between growing ice crystals becomes markedly higher than the average, inhibiting crystal growth and promoting the formation of smaller ice crystals. Additionally, oligosaccharides reduce the expansion of ice crystals and the formation of granule pores by lowering the frozen water content, thereby limiting ice crystal formation (Gunaratne et al., 2007). The inhibitory effect of oligosaccharides on starch granules followed the order: SUC > RAF > STA. This contradicts the findings of H Su, who reported that XOS, the oligosaccharide with the smallest molecular weight among FOS, GOS, and XOS, exhibited the least inhibition of pore formation. This discrepancy may be due to differences in the monosaccharide compositions of SUC and XOS. Moreover, although SUC has a smaller molecular weight, facilitating its entry into the starch granules, XOS is a mixture with a relatively higher molecular weight, which may hinder its entry into the granules.

### 3.4 The composition of frozen starch granules (damaged starch, protein, lipid)

With an increasing number of freeze-thaw cycles, the proportion of endogenous nutrients (protein and fat) within the granules decreased, but the damaged starch within the granules increased. This reduces the steric hindrance caused by amylopectin, leading to tighter aggregation of the starch molecular chains, an increase in double-helix formation, and a higher true density. These findings are consistent with those of previous studies. Compared to intact starch granules, damaged or cracked starch granules exhibit enhanced swelling pressure due to their increased water absorption capacity. This swelling pressure facilitates the leaching of chemical components from the cleaved starch residues. These results are consistent with those of Szymonska (Szymońska & Wodnicka, 2004), who demonstrated that the conversion of water to ice during freezing compresses starch granules, causing structural damage and the leaching of certain components.

The inclusion of oligosaccharides can mitigate the increase in damaged starch and the loss of endogenous nutrients (such as proteins and lipids). This effect may be attributed to the inhibition of ice crystal formation within the starch granules by oligosaccharides, thus reducing the swelling pressure induced by ice crystals and minimizing the leaching of endogenous nutrients due to starch degradation. Additionally, oligosaccharides can restrict starch regeneration, further reducing starch damage, as observed by Smits et al. (Smits et al., 2003). However, the results indicate that oligosaccharides are less effective in preventing lipid loss during freezing (Table 1). Among the tested oligosaccharides, SUC demonstrated superior performance in preserving endogenous nutrient components and preventing starch damage compared with STA and RAF. This finding aligns with that of Hou et al. (Hou et al., 2020), who suggested that smaller, more flexible sugars are less affected by steric hindrance, thereby providing better protein stabilization. The retention of endogenous nutrients plays a crucial role in limiting granule swelling, which, in turn, reduces the peak viscosity and recrystallization during thawing.

### 3.5 Amylose content of frozen starch

High amylose-containing starches are more easily to form reassembled ordered structure and reassociated gel networks containing stable double helices and strong crystallites during retrogradation and digestion (Chi et al., 2021). Therefore, starches with a high amylose content are more susceptible to retrogradation. Compared with NWS, the freeze-thaw treatment showed a reduction in starch amylose content (Table 1), which is consistent with the results of Section 3.4. Due to the possible rearrangement and aggregation of reactions between amylose and amylopectin, as well as between amylose molecules, resulting in a decrease in detectable free amylose content. After the addition of oligosaccharides, the amylose content of the starch-oligosaccharide system was significantly reduced (*p* < 0.05), indicating that oligosaccharides could reduce the amylose content in the starch system and delay the retrogradation of starch. This may be because oligosaccharides interact with amylose, making it easier to form a stable structure inside the starch granules and less likely to dissolve, and the amylose content may be reduced. For different oligosaccharides, the degree of amylose reduction was different, and the largest reduction degree was the starch granules with STA.

### 3.6 The surface morphology of frozen starch granules

**Fig. 1:**
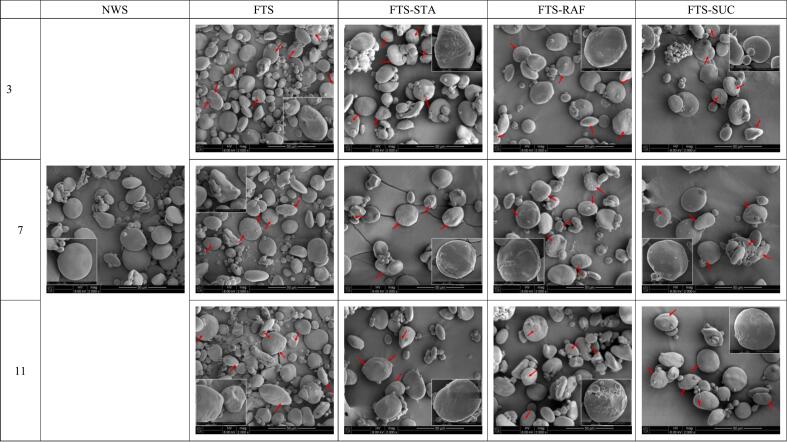
SEM images of particle morphology of native wheat starch and starch-oligosaccharides at different freezing-thaw cycles. (NWS: native wheat starch; FTS: freezing/thawing-treated wheat starch; FTS-STA: freezing/thawing-treated wheat starch with stachyose; FTS-RAF: freezing/thawing-treated wheat starch with raffinose; FTS-SUC: freezing/thawing-treated wheat starch with sucrose.)

The starch granules of NWS appeared disc-shaped with smooth surfaces. In contrast, the granules of FTS were spherical or elliptical, exhibiting grooves, depressions, and rough, porous surfaces, particularly in large starch granules (Fig. 1). This morphological change can be attributed to the physical pressure exerted on the granules by ice formation during freezing, which alters the surface microstructure. The pores observed on the surface of the granules are likely the result of the continuous formation and melting of ice crystals inside and outside the starch granules. The pores primarily arise from ice crystal growth, while the surrounding matrix reflects starch retrogradation induced by repeated freeze-thaw cycles (Klinmalai et al., 2024). The mechanical forces exerted on starch granules during freezing expand the internal channels of the starch molecules, facilitating the dissolution of more soluble substances. Additionally, moisture redistribution through the formed pores contributes to the concave appearance of the granules (Szymońska & Wodnicka, 2004).

Upon the introduction of the three oligosaccharides, damage to the starch granules was reduced compared to that in FTS, further confirming that oligosaccharides can lower the freezing point of the starch solution, resulting in smaller ice crystal formation. Most of the granules reverted to a disc-like shape. Although the surface roughness of the granules increased relative to that of NWS, it was less pronounced than that of FTS, with fewer depressions and less overall damage.

### 3.7 Long-range and short-range ordered structure of starch granules

#### 3.7.1 The X-ray diffraction of frozen starch granules (long range ordered structure)

**Fig. 2:**
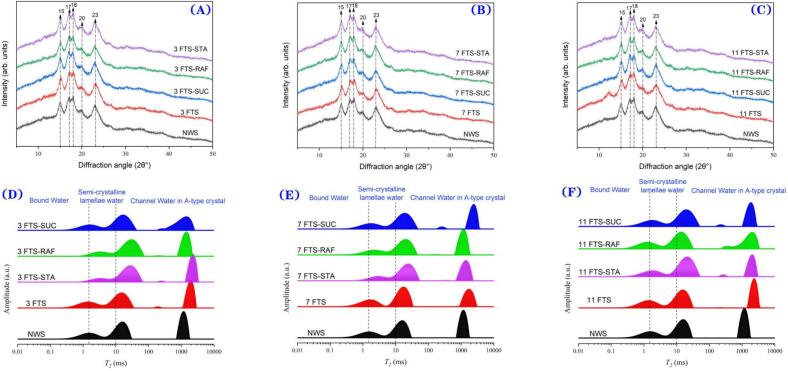
XRD patterns, moisture distribution of native wheat starch and starch-oligosaccharides at different freezing-thawing cycles. The XRD results of NWS group were derived from all results obtained in the reference NWS group of our study (Su et al., 2020). XRD:(A)3 FTS; (B)7 FTS; (C)11 FTS. Moisture distribution: (D)3 FTS; (E)7 FTS; (F)11 FTS. (NWS: native wheat starch; FTS: freezing/thawing-treated wheat starch; FTS-STA: freezing/thawing-treated wheat starch with stachyose; FTS-RAF: freezing/thawing-treated wheat starch with raffinose; FTS-SUC: freezing/thawing-treated wheat starch with sucrose.)

The double helix arrangement, stacking, and orientation of natural starch molecules can form monoclinic or hexagonal unit cells, which are arranged into well-organized and closely packed lattices, ultimately creating a highly ordered crystal structure referred to as a long-range crystal structure. Depending on the internal molecular configuration, the crystallization regions can be categorized as non-crystalline (characterized by diffuse diffraction peaks), sub-crystalline (with diffused diffraction peaks), and microcrystalline regions (which exhibit sharp diffraction peaks). X-ray diffraction (XRD) is an effective technique for assessing the crystallization type of starch and the regularity of its three-dimensional spatial structure (S. Wang, Li, et al., 2015). Wheat starch exhibited typical A-type diffraction peaks (15.4°, 17.0°, 18.0°, 20.0°, and 23.0°) before and after the freeze-thaw cycles (Fig. 2 A-C). No new peaks emerged in any of the samples, suggesting that starch did not undergo any chemical reactions or form new chemical bonds during the freeze-thaw process, even with the addition of oligosaccharides. The crystallization region of NWS is primarily composed of amylose double-helix structures. Upon freezing, the ice crystals disrupted the natural crystal structure, increasing the free water content, which facilitated the formation of helical structures between the starch molecular chains. During thawing, the molecular chains gradually reorganized into an initial crystalline structure consisting of smaller linear granules that were connected by hydrogen bonds. However, these granules exhibited broadened amorphous diffusion peaks, indicating a sub-microcrystalline structure.

The proportion of the FTS crystallization region increased, whereas that of the non-crystalline region decreased (Table 2). As the number of freeze-thaw cycles increased, the crystallinity of the starch also increased. This phenomenon may be attributed to the enhanced likelihood of forming restructured double helices with each freeze-thaw cycle, leading to a more ordered and stable double helix structure, which consequently increased the crystallinity. Notably, the proportion of sub-microcrystalline structures decreased after seven freeze-thaw cycles, possibly due to the significant damage caused by ice crystals to these structures. As the freeze-thaw cycles continued, the starch's regenerative ability likely overshadowed this negative effect. The addition of oligosaccharides did not alter the crystal structure of the system. However, compared to the FTS samples, the oligosaccharides reduced the proportion of the crystalline region. This reduction can be attributed to the competition between the hydroxyl groups of the oligosaccharide and starch molecules for hydrogen bonding interactions. When oligosaccharides are introduced into the starch system, they reduce the formation of hydrogen bonds between starch molecules, thereby weakening the interaction between starch molecules, making it difficult for starch chains to arrange in an orderly manner to form crystalline regions, which ultimately results in lower crystallinity (Kitagawa et al., 2025). The interaction between oligosaccharides and starch molecules altered the mobility and diffusion rate of starch molecules, making it more challenging for starch to find suitable positions for reorganization during crystallization. This inhibition of molecular rearrangement further contributed to the reduction in crystallinity. Moreover, sugar molecules in the non-crystalline region of starch granules can form crosslinking bonds (i.e., “sugar bridges”) between starch chains. These bridges limit starch swelling, reduce the swelling degree, and stabilize the crystallization region (Heng et al., 2021). The inhibitory effect of the oligosaccharides on crystallinity followed the order: STA > RAF > SUC. This trend is likely due to the hydrophilic properties of oligosaccharides, which are related to the number of hydroxyl groups. Oligosaccharides with a higher number of hydroxyl groups form stronger intermolecular hydrogen bonds with water molecules, resulting in increased hydrophilicity and water absorption. This is consistent with the findings of Tao et al. (Tao, Wang, Ali, et al., 2016).

**Table 2:** XRD pattern, selected FTIR peak ratios characteristics and the FWHMs of Raman band at 480 cm^−1^ of native wheat starch and starch-oligosaccharides at different freezing-thawing cycles.

	Index	Cycles	NWS	FTS	FTS-STA	FTS-RAF	FTS-SUC
XRD	Crystal pattern		A-type	A-type	A-type	A-type	A-type
	Proportion of the micro-crystalline region(%)	3	30.91 ± 0.05^a^	31.11 ± 0.09^b^	25.68 ± 0.07^c^	26.01 ± 0.11^c^	27.02 ± 0.07^d^
		7		32.06 ± 0.07^b^	25.96 ± 0.05^c^	26.75 ± 0.06^d^	28.36 ± 0.05^e^
		11		32.37 ± 0.02^b^	29.55 ± 0.09^c^	30.76 ± 0.04^d^	32.00 ± 0.08^b^
	Proportion of the sub-crystalline region(%)	3	28.25 ± 0.20^a^	32.69 ± 0.18^b^	32.88 ± 0.19^a^	38.21 ± 0.22^b^	32.72 ± 0.15^c^
		7		30.77 ± 0.15^b^	36.49 ± 0.22^c^	33.91 ± 0.03^d^	30.74 ± 0.08^e^
		11		34.92 ± 0.17^b^	35.95 ± 0.24^c^	34.01 ± 0.14^d^	25.28 ± 0.21^e^
	Proportion of the none-crystalline region (%)	3	40.85 ± 0.21^a^	36.20 ± 0.09^b^	41.44 ± 0.19^c^	35.78 ± 0.21^d^	40.27 ± 0.07^c^
		7		37.17 ± 0.23^b^	37.55 ± 0.21^b^	39.34 ± 0.11^c^	40.90 ± 0.16^a^
		11		32.71 ± 0.12^b^	34.50 ± 0.08^c^	35.23 ± 0.21^d^	42.72 ± 0.05^e^
FTIR	*R*_*1047/1022*_	3	0.979 ± 0.004^a^	0.973 ± 0.004^b^	0.911 ± 0.003^c^	0.946 ± 0.004^d^	0.963 ± 0.006^e^
		7		0.983 ± 0.006^b^	0.939 ± 0.001^b^	0.960 ± 0.006^b^	0.966 ± 0.006^c^
		11		1.002 ± 0.003^b^	0.945 ± 0.003^c^	0.985 ± 0.006^b^	0.986 ± 0.000^b^
	*R*_*995/1022*_	3	0.962 ± 0.003^a^	0.959 ± 0.003^b^	0.985 ± 0.006^a^	0.953 ± 0.003^b^	0.963 ± 0.004^bc^
		7		0.977 ± 0.005^b^	0.972 ± 0.004^b^	0.98 ± 0.005^c^	0.987 ± 0.005^c^
		11		0.979 ± 0.006^b^	0.956 ± 0.004^c^	0.969 ± 0.003^d^	0.975 ± 0.004^e^
Raman	FWHM of the band at 480 cm^−1^	3	18.11 ± 0.06^a^	18.82 ± 0.07^b^	19.81 ± 0.01^c^	19.26 ± 0.01^d^	18.9 ± 0.03^b^
		7		17.24 ± 0.07^b^	19 ± 0.04^c^	18.71 ± 0.04^d^	18.44 ± 0.05^e^
		11		15.95 ± 0.03^b^	18.5 ± 0.03^c^	18.35 ± 0.03^d^	18.15 ± 0.02^a^

Mean of three measurements ± standard deviation.Values for the same processing conditions and different additive groups with different letters are significantly different (*p* < 0.05). The XRD, FTIR results of NWS group were derived from all results obtained in the reference NWS group of our study (Su et al., 2020). (NWS: native wheat starch; FTS: freezing/thawing-treated wheat starch; FTS-STA: freezing/thawing-treated wheat starch with stachyose; FTS-RAF: freezing/thawing-treated wheat starch with raffinose; FTS-SUC: freezing/thawing-treated wheat starch with sucrose.)

#### 3.7.2 The fourier transform infrared spectroscopy and Raman spectroscopy of frozen starch granules (short-range ordered structure)

The absorption peak at 1047 cm-1 is typically associated with the crystalline region of starch, whereas the peak at 1022 cm-1 corresponds to the non-crystalline structure. Additionally, the absorption peak at 995 cm-1 is indicative of the ordered starch phase (Tongtong et al., 2022). Consequently, the ratio *R*_*1047/1022*_ reflects the relative content of the crystalline and non-crystalline regions of starch, with a larger ratio indicating a higher crystalline content. Similarly, the ratio *R*_*995/1022*_ represents the molecular order within the starch (Chen et al., 2017), where an increased ratio correlates with a greater content of the ordered phase. Furthermore, the short-range ordered structure of starch can be assessed using the half-width and intensity of the 480 cm^−1^ Raman peak. A smaller half-width at 480 cm^−1^ signifies a more closely packed crystal structure, thus increasing the order of the starch (S. Wang, Wang, et al., 2015). Higher crystallization and order in starch can accelerate ageing, leading to firmer, drier food with a degraded taste and shorter shelf life.

Compared to NWS, the *R*_*1047/1022*_ and *R*_*995/1022*_ ratios of the frozen starch did not show significant increases at low freeze-thaw cycles (*p* > 0.05). However, as the number of freeze-thaw cycles increased, both ratios exhibited an upward trend. This increase in the *R*_*1047/1022*_ and *R*_*995/1022*_ ratios after freezing may be attributed to the steric hindrance of amylose before freezing. Ice crystals can disrupt the starch structure, leading to amylose leaching from the granules. Upon thawing, amylopectin forms an intramolecular double helix, which subsequently reorganizes to establish a short-range ordered structure. With an increasing number of freeze-thaw cycles, the half-width at 480 cm^−1^ significantly decreased (*p* < 0.05), indicating an enhancement in the degree of short-range order. This is mainly due to the fracture or dissociation of glycosidic bonds in the starch molecule and hydrogen bonds in the starch crystallization region under the action of mechanical force, as observed through Fourier-transform infrared spectroscopy (FTIR), which corroborates the findings of Wang et al. (H. Wang et al., 2020).

In comparison to FTS, the half-width at 480 cm^−1^ increased significantly upon the addition of various oligosaccharides (*p* < 0.05). Additionally, the *R*_*1047/1022*_ ratio was significantly reduced (*p* < 0.05), consistent with the findings of Tao et al. (Tao et al., 2015). The observed crystallization inhibition effects were ranked as follows: STA > RAF > SUC. STA not only converts the starch crystalline regions into non-crystalline areas but also facilitates the absorption of more water, leading to the formation of larger ice crystals that disrupt initial amylopectin crystallization. Furthermore, STA contains more hydroxyl groups, which form stronger hydrogen bonds with starch molecules, effectively interfering with the hydrogen bonding between starch molecules and preventing aggregation and crystallization. The addition of oligosaccharides resulted in similar trends in the *R*_*1047/1022*_, *R*_*995/1022*_, and XRD analyses. These findings indicate that the changes in the number of double helices before and after freezing govern the short- and long-range ordered structures of the starch crystallization region, highlighting the significant impact of oligosaccharides on the starch double helix structure.

### 3.8 The water distribution of frozen starch granules (LF-NMR)

Low-field nuclear magnetic resonance (LF-NMR) is a valuable tool for assessing the moisture content, distribution, and migration processes of frozen starch granules. In NWS starch, three distinct water compartments have been identified by the starch granules, which are classified as bound water, water in the semi-crystalline lamellae, and channel water in the crystalline regions. By measuring the lateral relaxation time (*T*_*2*_), we can evaluate the binding states of starch and moisture, as well as the water distribution and retention capacity of the starch. Following the freeze-thaw treatment of NWS, the proportion of bound water and semi-crystalline lamellae water increased, whereas the proportion of channel water decreased (Fig. 2 D-F) (Table 3).

**Table 3:** Water distribution of native wheat starch and starch-oligosaccharides at different freezing-thawing cycles.

Index		Cycles	NWS	FTS	FTS-STA	FTS-RAF	FTS-SUC
LF-NMR	Proportion of bound water (%)	3	20.81 ± 0.08^a^	20.99 ± 0.18^a^	11.36 ± 0.01^b^	13.28 ± 0.06^c^	18.91 ± 0.08^d^
		7		23.00 ± 0.25^b^	13.98 ± 0.08^c^	16.75 ± 0.13^d^	19.27 ± 0.01^e^
		11		23.85 ± 0.15^b^	19.48 ± 0.03^c^	20.9 ± 0.03^d^	20.65 ± 0.03^e^
	Proportio of semi-crystallie lamellae water (%)	3	41.67 ± 0.21^a^	42.22 ± 0.14^b^	50.02 ± 0.25^c^	49.84 ± 0.14^c^	44.14 ± 0.1^d^
		7		46.04 ± 0.16^b^	49.96 ± 0.21^c^	44.67 ± 0.04^d^	45.78 ± 0.03^e^
		11		42.99 ± 0.30^b^	51.87 ± 0.15^c^	42.68 ± 0.05^d^	46.27 ± 0.01^e^
	Proportion of channel water in-type crystal (%)	3	37.52 ± 0.16^a^	36.79 ± 0.10^b^	38.62 ± 0.1^c^	36.88 ± 0.04^b^	36.94 ± 0.09^b^
		7		30.95 ± 0.33^b^	36.06 ± 0.09^c^	38.59 ± 0.01^d^	34.95 ± 0.11^e^
		11		33.17 ± 0.21^b^	28.65 ± 0.02^c^	36.42 ± 0.09^d^	33.08 ± 0.01^b^

Mean of three measurements ± standard deviation.Values for the same processing conditions and different additive groups with different letters are significantly different (*p* < 0.05). (NWS: native wheat starch; FTS: freezing/thawing-treated wheat starch; FTS-STA: freezing/thawing-treated wheat starch with stachyose; FTS-RAF: freezing/thawing-treated wheat starch with raffinose; FTS-SUC: freezing/thawing-treated wheat starch with sucrose.)

The addition of the three oligosaccharides significantly reduced the proportion of bound water in the wheat starch granules (*p* < 0.05). This is attributed to the competitive interaction between the hydroxyl groups of the oligosaccharides and those on the starch surface, which bind more effectively to water. Consequently, the hydrogen bonding between the starch hydroxyl groups and water molecules is diminished, thereby reducing the mobility of the bound water molecules. This disruption prevents water from being incorporated into the starch recrystallization structure, thereby lowering the proportion of bound water. Among the three oligosaccharides, STA exhibited the highest hydration capacity. This is due to its larger molecular size and the presence of more equatorial hydroxyl groups, which form stronger intermolecular hydrogen bonds with water molecules. Consequently, STA demonstrates higher hydrophilicity and water absorption than RAF and SUC (Hedayati et al., 2016). Furthermore, the steric hindrance caused by STA molecules on the surface of the starch granules prevents efficient water binding, further decreasing the bound water proportion.

### 3.9 The thermal property of frozen starch granules

The thermal and phase transition characteristics of wheat starch were analyzed using Differential Scanning Calorimetry (DSC). Key parameters, including the gelatinization transition temperatures *(T*_*0*_, *T*_*p,*_ and *T*_*c*_) and enthalpy change (*ΔH*), were evaluated*.* The parameter *T*_*0*_ reflects the energy required to break the helix-helix structure, providing insights into the thermal stability of the ordered molecular structure. An increase in *T*_*0*_ is indicative of a denser helical structure, which corresponds to more perfect crystalline regions and a larger granule size. *ΔT* (*T*_*c*_-*T*_*0*_), which represents the uniformity of the ordered molecular structure of starch (Xia et al., 2024) is smaller when the starch granules exhibit a more uniform grain distribution. Both the initial gelatinization temperature (*T*_*0*_) and *ΔH* of the starch increased with an increase in the number of freeze-thaw cycles, whereas the crystallization temperature *Tc* decreased (Table 4). This led to a reduction in *ΔT* (*Tc*-*T*_*0*_), suggesting that freeze-thaw cycles affect the uniformity of the granules. During freezing, ice crystals disrupt the natural lamellar crystal structure of starch, and the recrystallized lamellar layers formed during thawing and regeneration may be more uniform. Furthermore, *ΔH* is positively correlated with the number of amylopectin grains, short-range molecular sequences and double helix segments (Vandeputte et al., 2003). Starch granules with highly ordered crystalline regions exhibit increased resistance to gelatinization, as evidenced by their higher *ΔH* values and elevated gelatinization temperatures. The observed increase in starch gelatinization is likely due to the hydrogen bonds formed during the cooling of the starch paste. At higher heating temperatures, the energy required to break the regenerated double helix exceeds that required to break the natural starch structure.

**Table 4:** Gelatinization parameters and pasting properties of native wheat starch and starch-oligosaccharides at different freezing-thawing cycles.

	Index	Cycles	NWS	FTS	FTS-STA	FTS-RAF	FTS-SUC
*DSC*	*T*_*o*_ (°C)	3	60.43 ± 0.18^a^	63.33 ± 0.15^b^	59.82 ± 0.2^b^	59.25 ± 0.2^a^	60.33 ± 0.17^b^
		7		63.95 ± 0.15^b^	61.45 ± 0.17^c^	60.46 ± 0.2^d^	61.75 ± 0.12^c^
		11		63.94 ± 0.17^b^	59.93 ± 0.17^c^	61.6 ± 0.05^d^	60.12 ± 0.06^c^
	*T*_*p*_ (°C)	3	65.02 ± 0.15^a^	68.25 ± 0.18^b^	63.78 ± 0.16^a^	63.7 ± 0.22^a^	64.59 ± 0.15^c^
		7		67.44 ± 0.02^b^	65.92 ± 0.22^c^	64.72 ± 0.16^d^	65.99 ± 0.23^c^
		11		65.62 ± 0.19^b^	64.61 ± 0.23^c^	65.92 ± 0.17^d^	64.2 ± 0.22^c^
	*T*_*c*_ (°C)	3	69.69 ± 0.19^a^	72.75 ± 0.14^b^	68.28 ± 0.2^a^	67.81 ± 0.2^c^	68.3 ± 0.01^a^
		7		71.20 ± 0.15^b^	69.68 ± 0.24^c^	68.89 ± 0.17^d^	70.72 ± 0.21^e^
		11		70.52 ± 0.17^b^	68.5 ± 0.13^a^	70.14 ± 0.15^c^	68.54 ± 0.12^a^
	Δ*H* (J/g)	3	9.82 ± 0.03^a^	9.90 ± 0.03^b^	7.40 ± 0.01^c^	7.51 ± 0.03^d^	7.92 ± 0.02^e^
		7		10.01 ± 0.02^b^	7.76 ± 0.03^c^	8.27 ± 0.02^d^	8.47 ± 0.03^e^
		11		10.24 ± 0.04^b^	8.25 ± 0.02^c^	8.59 ± 0.03^d^	8.75 ± 0.02^e^
RVA	Pasting temperature(°C)	3	92.95 ± 0.18^a^	92.05 ± 0.17^b^	92.05 ± 0.23^c^	91.69 ± 0.29^b^	93.95 ± 0.24^c^
		7		92.32 ± 0.22^b^	92.98 ± 0.29^c^	92.63 ± 0.29^c^	91.66 ± 0.32^b^
		11		92.26 ± 0.03^b^	92.92 ± 0.31^b^	93.76 ± 0.08^c^	93.75 ± 0.12^d^
	Peak viscosity(cP)	3	1499.52 ± 5.93^a^	1654.93 ± 5.06^b^	1390.38 ± 4.22^c^	1321.34 ± 2.77^d^	1279.08 ± 3.14^c^
		7		1666.32 ± 3.65^b^	1414.98 ± 1.54^c^	1383.75 ± 1.21^c^	1290.62 ± 3.96^c^
		11		1778.14 ± 4.61^b^	1587.95 ± 0.85^c^	1437.11 ± 4.18^d^	1381.99 ± 2.97^d^
	Breakdown viscosity (cP)	3	517.36 ± 1.62^a^	480.84 ± 1.07^b^	257.99 ± 0.26^c^	261.28 ± 0.83^d^	283.07 ± 0.94^c^
		7		524.99 ± 1.84^b^	250.27 ± 0.93^c^	311.94 ± 0.59^d^	308.53 ± 0.61^c^
		11		607.69 ± 1.53^b^	331.51 ± 0.91^c^	283.23 ± 0.77^c^	307.13 ± 1.59^d^
	Setback viscosity (cP)	3	901.27 ± 2.38^a^	946.97 ± 1.19^b^	496.19 ± 1.92^c^	563.91 ± 1.94^d^	566.46 ± 1.52^c^
		7		967.41 ± 2.54^b^	600.96 ± 1.78^c^	605.62 ± 1.75^d^	616.17 ± 1.88^c^
		11		1026.84 ± 3.57^b^	640.21 ± 1.67^c^	662.76 ± 1.06^c^	707.06 ± 1.54^d^

Mean of three measurements ± standard deviation. Values for the same processing conditions and different additive groups with different letters are significantly different (*p* < 0.05). The results of NWS group were derived from all results obtained in the reference NWS group of our study (Su et al., 2020). (NWS: native wheat starch; FTS: freezing/thawing-treated wheat starch; FTS-STA: freezing/thawing-treated wheat starch with stachyose; FTS-RAF: freezing/thawing-treated wheat starch with raffinose; FTS-SUC: freezing/thawing-treated wheat starch with sucrose.)

Compared to FTS, the addition of different oligosaccharides significantly reduced both *T*_*0*_ and *ΔH* (*p* < 0.05), likely due to the influence of oligosaccharides on the swelling behavior of starch granules before gelatinization. Conversely, the interaction between water and oligosaccharides reduces the moisture activity, thereby enhancing starch plasticity. In contrast, oligosaccharides readily incorporate into the starch molecular structure, potentially limiting the leaching of amylose and endogenous nutrients while increasing the steric hindrance of amylopectin chain rearrangements during thawing. Consequently, the energy required for gelatinization decreases, resulting in a *ΔH* value that is more closely aligned with starch crystallinity. The effects of different oligosaccharides on *ΔH* followed the order: STA > RAF > SUC. This can be attributed to the type of glycosidic bonds present in the oligosaccharides, which influence their interactions with starch. Sucrose consists of *α*-1,2-glycosidic bonds between *d*-glucose and *β-D-fruct*. Raffinose has a single *D*-galactoside unit linked to sucrose through an *α*-1,4-glycosidic bond, whereas STA has a single *D*-galactosyranosyl unit linked to raffinose via an *α*-1,4-glycosidic bond. The helical structure formed by hydrogen bonding in STA tends to reduce the hydration capacity of sugar and destabilize the water structure. Consequently, the inhibitory effect of STA was almost identical to that of raffinose.

### 3.10 The viscosity measurement of frozen starch granules

Starch gelatinization plays a pivotal role in the texture and structure of various baked goods, including cakes, bread, and muffins. However, excessive gelatinization can negatively affect the quality of certain products, including sugar cookies and shortbread (Tao et al., 2015). The viscosity characteristics during gelatinization are influenced by factors such as granule swelling, the proportion of swollen granules, and the magnitude of their interactions. Other key determinants include amylose leaching, starch crystallinity, granule swelling, and degree of gelatinization. Additives during gelatinization can significantly affect granule swelling and paste viscosity properties, with various additives promoting or inhibiting amylose leaching. Amylose, in particular, has been shown to restrict the swelling of starch granules.

The gelatinization temperature of FTS was lower than that of NWS (Table 4). This decrease is attributed to the disruption of the starch crystal structure by frozen ice crystals, which reduces the energy required for gelation and lowers the gelatinization temperature. The gelatinization temperature of FTS increased with the addition of different oligosaccharides. This can be explained by the ability of oligosaccharides to limit amylose leaching, thereby restricting the swelling of starch granules, as previously reported by Zi et al. (Zi et al., 2019). In the frozen starch system, highly hydrophilic oligosaccharides interact with water molecules, restricting a significant portion of the free water and reducing the water activity within the system. Reduced water activity means that less free water is available for starch gelatinization, which consequently limits the water absorption and swelling of starch granules. Consequently, higher temperatures are required to achieve the moisture conditions necessary for gelatinization, thereby increasing the gelatinization temperature.

Peak viscosity is closely associated with the swelling capacity of starch granules, reflecting the viscosity observed when the granules are completely gelatinized (Zavareze & Dias, 2010). The primary factor influencing starch granule swelling is amylopectin, whereas amylose and lipids inhibit it. FTS exhibited a higher peak viscosity than NWS, with an increase observed as the number of freeze-thaw cycles increased (Table 4). After adding oligosaccharides, the peak viscosity of starch was significantly reduced (*p* < 0.05). These oligosaccharides demonstrated a stronger ability to interact with water than starch, leading to reductions in both peak viscosity and starch swelling. This, in turn, may decrease the water content required for gelatinization of starch. Among the tested oligosaccharides, SUC exhibited the greatest capacity to reduce peak viscosity, followed closely by RAF and STA.

The breakdown viscosity measures the ability of starch granules to resist shear forces during the heating process. A lower breakdown viscosity indicates that the starch can better withstand shear stress without undergoing degradation (Table 4). The increase in breakdown viscosity with the number of freeze-thaw cycles can be attributed to the disruption of the NWS crystal structure and a corresponding increase in the swelling capacity of the granules, which ultimately reduces the stability of the starch paste. However, after the addition of STA, RAF, and SUC, the breakdown viscosity decreased, suggesting that oligosaccharides can prevent the degradation of starch granules, material dissolution, and swelling during the freeze-thaw cycles. The inhibitory effect was most pronounced with STA, followed by RAF and SUC. STA's larger molecular weight (Mw) and the greater number of hydroxyl groups may contribute to its stabilizing effect on starch granules.

Regeneration is a complex process involving structural rearrangements that depend on the degree of structural damage incurred during heating and the extent of the remaining short-range order (C. & J., 2022). Factors such as molecular mobility, amylose interference, amylose recrystallization, storage conditions, and various intrinsic and extrinsic conditions influence starch retrogradation (S. Wang, Li, et al., 2015). Setback viscosity, defined as the difference between valley viscosity and final viscosity during the cooling stage, is related to the formation of a gel network and involves amylose and long amylopectin chain formation. The higher the amylose content, the greater the regeneration viscosity due to the rapid rearrangement of amylose during cooling (Zobel, 1988). Following freeze-thaw treatment, amylose extracted from wheat starch reorganizes into a single helical structure, leading to an increase in setback viscosity (Table 4), which is consistent with the changes observed in peak viscosity and XRD data. The setback viscosity increased with the number of freeze-thaw cycles. However, after the addition of oligosaccharides, the setback viscosity was significantly reduced (*p* < 0.05). This reduction is due to the interaction between oligosaccharides and water, which decreases the water content required for starch regeneration and limits the leaching of endogenous nutrients. The steric hindrance imparted by the oligosaccharide structures prevents the aggregation and helicity of starch molecular chains, significantly reducing the viscosity of the regenerated starch (*p* < 0.05). Among the oligosaccharides, STA was the most effective at reducing the setback viscosity of wheat starch.

### 3.11 The rheological properties of frozen starch paste

#### 3.11.1 Steady rheological properties of frozen starch paste

**Fig. 3:**
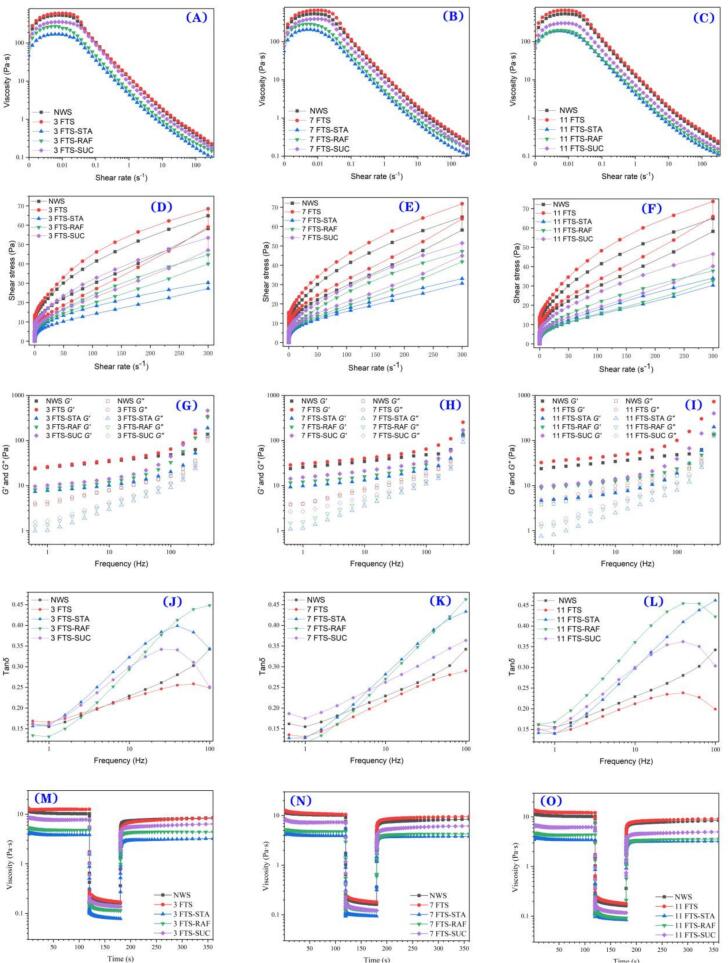
Rheological properties of (A-C) apparent viscosity, (D—F) shear stress, (G-I) storage modulus *G*′ and loss modulus *G*″, (J-L) tan δ, (M-O) In-shear structural recovery for the wheat starch paste that interacting with three oligosaccharides at different freezing-thaw cycles. (NWS: native wheat starch; FTS: freezing/thawing-treated wheat starch; FTS-STA: freezing/thawing-treated wheat starch with stachyose; FTS-RAF: freezing/thawing-treated wheat starch with raffinose; FTS-SUC: freezing/thawing-treated wheat starch with sucros.

The rheological characteristics of starch are key indicators of its properties, providing insights into the changes in dough viscoelasticity during freezing and storage. As shown in Fig. 3A-C, the apparent viscosity of all starch samples decreased as the shear rate increased, with a flow characteristic index (*n*) of less than 1 (Table 5). This suggests that the adsorbed starch paste exhibited pseudoplastic behavior, behaving as a non-Newtonian fluid with shear-thinning properties. This shear-thinning behavior can be attributed to the extensive breakdown of intramolecular and intermolecular bonds within the starch network micelles caused by high-speed shearing (Bhandari et al., 2002). The power-law model provided a good fit for the measured viscosity changes (*R*^2^ ≈ 1).

**Table 5:** The flow behavior curve and in-shear structure recovery parameters of native wheat starch and starch-oligosaccharides at different freezing-thawing cycles.

	Index	Cycles	NWS	FTS	FTS-STA	FTS-RAF	FTS-SUC
Up-sweep curve	*τ*_*0*_ (Pa)	3	5.723 ± 0.716^a^	5.914 ± 0.752^a^	1.842 ± 0.219^b^	3.009 ± 0.280^c^	4.864 ± 0.576^a^
		7		6.969 ± 0.877^a^	2.127 ± 0.210^b^	2.921 ± 0.254^c^	3.696 ± 0.367^d^
		11		6.949 ± 0.836^a^	2.186 ± 0.207^b^	2.191 ± 0.236^b^	3.508 ± 0.386^c^
	*K* (Pa•s*n*)	3	4.807 ± 0.716^a^	5.429 ± 0.762^a^	1.999 ± 0.208^b^	1.633 ± 0.205^b^	2.987 ± 0.517^c^
		7		5.326 ± 0.879^a^	1.431 ± 0.168^b^	1.661 ± 0.180^b^	2.301 ± 0.296^c^
		11		5.307 ± 0.826^a^	1.191 ± 0.149^b^	1.970 ± 0.203^c^	2.547 ± 0.344^d^
	*n*	3	0.433 ± 0.028^ac^	0.426 ± 0.026^a^	0.458 ± 0.019^ac^	0.562 ± 0.023^b^	0.483 ± 0.032^c^
		7		0.432 ± 0.031^a^	0.530 ± 0.022^b^	0.574 ± 0.020^c^	0.526 ± 0.024^b^
		11		0.439 ± 0.029^a^	0.568 ± 0.023^b^	0.501 ± 0.019^c^	0.487 ± 0.025^d^
	*R*^*2*^	3	0.963 ± 0.002	0.966 ± 0.002	0.983 ± 0.001	0.982 ± 0.000	0.957 ± 0.001
		7		0.955 ± 0.002	0.982 ± 0.001	0.987 ± 0.001	0.979 ± 0.002
		11		0.96 ± 0.002	0.982 ± 0.001	0.985 ± 0.002	0.974 ± 0.001
Down-sweep curve	*τ*_*0*_ (Pa)	3	4.639 ± 0.243^a^	4.152 ± 0.242^b^	1.767 ± 0.105^c^	2.806 ± 0.162^d^	3.389 ± 0.201^e^
		7		5.165 ± 0.278^a^	2.117 ± 0.111^b^	2.663 ± 0.181^c^	3.056 ± 0.193^c^
		11		5.053 ± 0.290^a^	2.125 ± 0.115^b^	1.987 ± 0.143^b^	2.814 ± 0.176^c^
	*K* (Pa•s*n*)	3	2.146 ± 0.185^a^	1.066 ± 0.124^b^	1.058 ± 0.081^b^	1.265 ± 0.113^c^	1.292 ± 0.131^c^
		7		2.419 ± 0.213^a^	1.286 ± 0.089^b^	0.945 ± 0.105^c^	1.137 ± 0.120^b^
		11		2.245 ± 0.211^a^	0.974 ± 0.081^b^	0.996 ± 0.098^b^	1.040 ± 0.111^b^
	*n*	3	0.550 ± 0.016^a^	0.678 ± 0.020^b^	0.545 ± 0.014^a^	0.579 ± 0.017^c^	0.603 ± 0.019^c^
		7		0.545 ± 0.016^a^	0.531 ± 0.013^a^	0.639 ± 0.021^b^	0.617 ± 0.020^b^
		11		0.564 ± 0.017^a^	0.576 ± 0.015^a^	0.585 ± 0.018^ab^	0.612 ± 0.020^b^
	*R*^*2*^	3	0.991 ± 0.003	0.989 ± 0.003	0.993 ± 0.001	0.991 ± 0.001	0.989 ± 0.002
		7		0.99 ± 0.001	0.994 ± 0.001	0.988 ± 0.002	0.989 ± 0.001
		11		0.99 ± 0.001	0.992 ± 0.002	0.989 ± 0.001	0.988 ± 0.002
Thixotropic loop Area (Pa/s)		3	2814.99 ± 6.96^a^	4620.43 ± 20.91^b^	1084.73 ± 2.66^c^	1338.16 ± 6.65^d^	2569.66 ± 6.59^e^
		7		3326.13 ± 21.69^b^	569.23 ± 8.15^c^	1917.87 ± 10.01^d^	2253.75 ± 12.64^e^
		11		3592.57 ± 20.70^b^	920.37 ± 2.45^c^	1701.15 ± 5.92^d^	2227.01 ± 8.08^e^

Mean of three measurements ± standard deviation. Values for the same processing conditions and different additive groups with different letters are significantly different (*p* < 0.05). (NWS: native wheat starch; FTS: freezing/thawing-treated wheat starch; FTS-STA: freezing/thawing-treated wheat starch with stachyose; FTS-RAF: freezing/thawing-treated wheat starch with raffinose; FTS-SUC: freezing/thawing-treated wheat starch with sucrose.)

The consistency index (*K*) of FTS showed an upward trend (Table 5), indicating that freeze-thawing disrupted the natural crystalline structure of starch, facilitating starch swelling. Conversely, the starch paste containing oligosaccharides exhibited a lower *K* value. This can be attributed to the ability of oligosaccharides to form crosslinks (sugar bridges) between starch chains in the non-crystalline regions of the starch granules, thereby limiting starch swelling and stabilizing these regions. Among the various samples, frozen wheat starch with added STA exhibited the lowest apparent viscosity, whereas frozen wheat starch with added SUC exhibited the highest. This difference may be due to STA's higher molecular flexibility and stronger hydrogen bonding potential, which enhance the formation of hydrogen bonds with starch chains, thereby restricting starch swelling during gelatinization.

#### 3.11.2 Dynamic rheological properties of frozen starch paste

The storage modulus (*G'*) was higher than the loss modulus (*G"*) (Fig. 3 G-I), indicating that the starch exhibited a robust gel structure. Following freeze-thaw treatment, both moduli increased, with the rate of increase becoming more pronounced as the number of freeze-thaw cycles increased. This enhancement is likely due to amylose regeneration and recrystallization. The addition of oligosaccharides led to a reduction in both *G'* and *G"*, which can be explained by the ability of oligosaccharides to elevate gelation and gelatinization temperatures. This delay in granule swelling alters the degree of granule expansion. Consequently, stronger interactions between starch paste molecules form a denser structure, reducing moisture migration, improving shear resistance, and enhancing its stability. Notably, the starch-containing STA exhibited the lowest *G'* and *G"* values, which may be due to the larger ice crystals formed by STA during the freeze-thaw cycles, coupled with the reduced permeability of the hydrophobic shells surrounding the starch granules. The resulting loosening of the starch matrix, which hinders the formation of a three-dimensional network, leads to a decrease in the *G'* of starch paste. Additionally, the limited swelling of the starch granules contributed to the reduction in *G"*.

The loss tangent (tan*δ*) serves as an indicator of starch gel rigidity, with lower tanδ values corresponding to higher rigidity (Fig. 3 J-L). The tan*δ* of FTS decreased, and as the number of freeze-thaw cycles increased, the magnitude of the decrease in tan*δ* increased. This suggests that freezing enhances the rigidity of the starch gel. This effect is likely due to the rearrangement of amylopectin and its increased interaction with the starch molecular chains, which together form a solid gel network during cooling, thereby enhancing gel rigidity. When the three oligosaccharides were added, tan*δ* was also increased, which is consistent with previous research (Liu et al., 2022). This can be attributed to the hydrogen bonding between oligosaccharides and the leached amylose, which may reduce the extent of amylose leaching and inhibit the formation of cross-linked hydrogen bonds, thus decreasing the viscoelasticity of the gel network (Tongtong et al., 2022). Interestingly, although SUC was most effective in preserving endogenous nutrient components, it resulted in the lowest tan*δ* value, a phenomenon that requires further investigation.

#### 3.11.3 In-shear structural recovery properties of frozen starch paste

The recovery rates for all pastes were below 1, indicating that the initial starch paste structure was disrupted under high shear conditions and that the new structure formed under low shear was weaker (Fig. 3 M-O). Among the samples, the FTS recovery rate exhibited a decreasing trend. This may be attributed to the shearing of swollen, gelled granules (non-recrystallized fractions) into smaller pieces after freeze-thawing under high shear, leading to a reduced shear strength. In contrast, the starch pastes containing STA and RAF exhibited higher recovery rates. The oligosaccharides and amylose limit the swelling of starch granules, and their interaction, along with an increase in starch rigidity, helps mitigate granule breakdown under high shear conditions (J., Erica, C., & J., 2022; Vamadevan & Bertoft, 2020).

### 3.12 Digestive resistance of frozen starch

The higher the RS or SDS content or the lower the RDS content means that the digestion and absorption in the human body is relatively slow (Ma et al., 2024). Compared with NWS, the RS of FTS was significantly higher (*p* < 0.05) (Table 6), which may be due to the fact that freeze-thaw destroys the original structure of starch, causing some starch molecules to rearrange to form a structure that is difficult to be affected by digestive enzymes, and some crystalline areas inside starch granules would become more stable. All of these factors have led to an increase in the proportion of RS, which indicates that freeze-thaw reduces the digestibility of starch. After the addition of oligosaccharides, the RS content still increased, which was contrary to the XRD results and may be due to the increase of enzyme contact sites with the enlarged pore size in starch granules. It is consistent with the findings of which showed that malto-oligosaccharides were produced after starch was fermented by lactic acid bacteria (Julien et al., 2012), while the RS content in starch increased. However, these changes are not absolute, and the ability of digested starch in the environment might depend on factors such as the type of oligosaccharides and starches, the processing conditions, and so on. Among the modified oligosaccharides, the starch system with SUC had the highest resistant starch content, and the starch system with STA added had the lowest resistant starch content, which was consistent with their inhibition effect on the crystallinity of starch granules, indicating that STA had the least effect on the increase of starch digestion resistance.

**Table 6:** The proportion of different fast, slow and resistant starch of wheat starch and starch-oligosaccharides at different freezing-thawing cycles.

Index		Cycles	NWS	FTS	FTS-STA	FTS-RAF	FTS-SUC
Digestive starch	RS(%)	3	13.99 ± 0.37^a^	75.14 ± 1.2^b^	80.43 ± 1.66^c^	82.47 ± 0.74^c^	83.53 ± 1.92^c^
		7		72.28 ± 1.64^b^	71.06 ± 1.90^b^	78.8 ± 1.07^c^	79.21 ± 1.19^c^
		11		53.94 ± 1.15^b^	69.02 ± 1.37^c^	58.42 ± 1.08^d^	59.24 ± 0.56^d^
	SDS(%)	3	75.9 ± 0.57^a^	1.57 ± 0.02^b^	6.89 ± 0.13^c^	6.05 ± 0.07^d^	15.61 ± 0.35^e^
		7		18.83 ± 0.12^b^	16.96 ± 0.40^c^	20.85 ± 0.50^d^	11.5 ± 0.43^e^
		11		18.1 ± 0.25^b^	19.69 ± 0.16^c^	32.49 ± 0.59^d^	28.9 ± 0.10^e^
	RDS(%)	3	10.11 ± 0.27^a^	23.29 ± 0.48^b^	12.68 ± 0.38^c^	11.48 ± 0.37^d^	0.86 ± 0.12^e^
		7		8.89 ± 0.17^b^	11.98 ± 0.06^c^	0.35 ± 0.32^d^	9.29 ± 0.21^b^
		11		27.96 ± 0.66^b^	11.29 ± 0.27^c^	9.09 ± 0.19^d^	11.86 ± 0.38^e^

Mean of three measurements ± standard deviation. Values for the same processing conditions and different additive groups with different letters are significantly different (*p* < 0.05). (NWS: native wheat starch; FTS: freezing/thawing-treated wheat starch; FTS-STA: freezing/thawing-treated wheat starch with stachyose; FTS-RAF: freezing/thawing-treated wheat starch with raffinose; FTS-SUC: freezing/thawing-treated wheat starch with sucrose.)

### 3.13 Analysis of the structural changes of starch granules under freeze-thaw cycles and the mechanism of action of prebiotic oligosaccharides

The internal channels of starch granules, which are weak points for free water and ice crystal formation, are subjected to compression and stress during the freeze-thaw cycles. This results in the formation of cake-like wedges in the regenerated and crushed granules (Wei et al., 2018). This phenomenon is corroborated by SEM images of wheat starch-oligosaccharide granules subjected to freeze-thaw treatment, where larger granules transition from round to oval shapes, and their surfaces become rougher. Continuous freeze-thaw cycles lead to the free migration of water and local aggregation, forming ice crystals that increase in size. These ice crystals generate pores and cracks on the surface and within the granules, resulting in an increase in the particle size, porosity, and starch damage rate. Protein and lipid losses further contribute to higher granule density, which is caused by damage to the starch structure after freeze-thawing. The non-crystalline portion escaped from the granules along with the interparticle water, which enhanced the crystallinity of the granules. As the number of freeze-thaw cycles increased, the crystallinity of the starch exhibited an upward trend, contrary to the findings of Kim et al. (Zhou et al., 2024). The observed differences may be due to the use of different starch types in these studies. The increase in crystallinity observed in this study can be attributed to the enhanced formation of reborn double helices during each successive freeze-thaw cycle. This resulted in more ordered and stable double helices, leading to higher crystallinity. Free water penetrates starch granules more readily, and the interactions between amylose-amylose chains and amylopectin-starch molecular chains become stronger. Nutrient loss further facilitates amylose regeneration. Fourier-transform infrared spectroscopy confirmed an increase in double-helix formation and short-range order, whereas X-ray diffraction results demonstrated the development of a more ordered long-range three-dimensional network structure. Additionally, the low-field NMR results, in conjunction with the increased water content, provided evidence for the formation of specific inverse-gradient starch crystal structures.

Three types of oligosaccharides can mitigate the adverse effects of freezing on wheat starch granules through several mechanisms: (1) Oligosaccharides with lower *T*_*g*_ reduce the freezing point of the solution, thereby minimizing the formation of large ice crystals in the free water. This inhibits the extrusion and starch granule aggregation and swelling pressure from ice crystals (van der Sman, 2016), thereby inhibiting the increase in the particle size of granules and alleviating starch granule damage and nutrient leaching (Smits et al., 2003). The reduced swelling pressure also hinders the rearrangement of amylopectin chains during thawing. (2) The hydroxyl groups of oligosaccharides compete with starch molecules to form hydrogen bonds, reducing the formation of water bonds between starch molecules. This weakens the molecular interactions of starch, preventing the ordered arrangement of starch chains necessary for crystallization, thereby decreasing the crystallinity of starch granules (Kitagawa et al., 2025). The interaction between water and oligosaccharides reduces the amount of frozen water in starch granules, thereby limiting structural shrinkage and preventing increases in porosity (Gunaratne et al., 2007). Moreover, this reduces the mobility of water molecules bound to sugar‑hydrogen bonds, preventing water incorporation into the starch recrystallization structure. (3) Oligosaccharides form crosslinking bonds (sugar bridges) within the non-crystalline region of starch granules, restricting swelling and stabilizing the crystallization region (Heng et al., 2021). (4) The strong hydrophilicity of oligosaccharides limits the availability of free water, thereby reducing water activity within the system (Hedayati et al., 2016). Reduced water activity prevents starch gelatinization by limiting the water absorption and swelling of starch granules, thereby increasing the temperature required for gelatinization.

Additionally, STA, RAF, and SUC helped maintain the gelatinization temperature of wheat starch during the freeze-thaw cycle, preventing starch gelatinization and reducing *ΔH*. This is particularly relevant for the quality of baked goods, and selecting the appropriate oligosaccharide type can help reduce the sweetener content while increasing the dietary fiber content. Among the oligosaccharides, there were also differences in the impact of different glycosidic bond types, polymerization degrees, and hydrophilicity on the frozen starch. SUC was the most effective at reducing particle size, granule porosity, alleviating starch damage, preserving endogenous nutrients, and peak viscosity, whereas STA was the most effective at reducing true density, crystallinity, bound water ratio, decomposition viscosity, and regeneration viscosity in wheat starch granules.

## 4 Conclusions

In the front-end freezing process of food, starch granules remain largely intact in frozen dough products; however, alterations to their structure significantly influence the product's subsequent properties. This study examined the morphological characteristics, endogenous nutrient components, gelatinization, and rheological properties of wheat starch granules and oligosaccharide–starch mixtures (STA, RAF, and SUC) across multiple freeze-thaw cycles. The results demonstrated a positive correlation between the number of freeze-thaw cycles and starch regeneration levels. Moreover, prebiotic oligosaccharides mitigated the starch structural damage caused by refrigeration. Following the freeze-thaw cycles, starch exhibited a more ordered recrystallization structure, resulting in enhanced short- and long-range molecular chain order. The incorporation of oligosaccharides effectively reduced the content of amylose in the starch system to slow down the retrogradation of starch, inhibited recrystallization during the thawing process, reducing both the energy storage modulus and loss modulus of the starch paste. Additionally, the presence of oligosaccharides decreased the porosity and regeneration of starch granules, likely due to the suppression of ice crystal expansion. Among the three oligosaccharides tested, STA exhibited the most pronounced inhibitory effect on starch regeneration, particularly in limiting the hydration capacity of the wheat starch granules. These findings suggest that oligosaccharides have significant potential to improve freezing stability, with their effects varying according to the specific oligosaccharide type. However, it had a promoting effect on the digestive resistance of starch. This study did not investigate the impact of prebiotic oligosaccharides on frozen dough containing treated starch granules. Future research should focus on elucidating the mechanical and regenerative mechanisms of frozen dough under such treatments.

## CRediT authorship contribution statement

**Juanjuan Guo:** Writing – review & editing, Writing – original draft, Funding acquisition, Data curation. **Zengming Huang:** Methodology, Conceptualization. **Peilin Chen:** Visualization, Data curation. **Xiantong Wu:** Formal analysis. **Xu Lu:** Supervision.

## Declaration of competing interest

The authors declare that they have no known competing financial interests or personal relationships that could have appeared to influence the work reported in this paper.

## Data Availability

No data was used for the research described in the article.

## Appendix A Supplementary data

Supplementary material: Raman spectra of native wheat starch and starch-oligosaccharides at different freezing-thaw cycles. Image 1
