# Supplementary material for: The changes in multi-scale structure and properties of wheat starch after interaction with oligosaccharides of different polymerization degrees under different freeze-thaw cycles
Source: Food Chem X. 2025 Apr 24;27:102482. doi: 10.1016/j.fochx.2025.102482 (PMC12084406; doi:10.1016/j.fochx.2025.102482)

**Fig S1**Raman spectra of native wheat starch and starch-oligosaccharides at different freezing-thaw cycles.

(NWS: native wheat starch; FTS: freezing/thawing-treated wheat starch; FTS-STA: freezing/thawing-treated wheat starch with stachyose; FTS-RAF: freezing/thawing-treated wheat starch with raffinose; FTS-SUC: freezing/thawing-treated wheat starch with sucrose.)


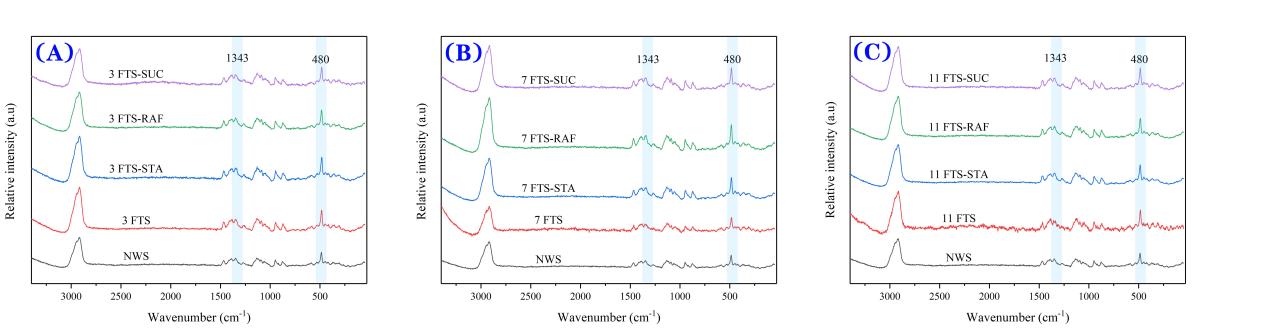

Supplement: Supplementary file 1 — Supplementary material: Raman spectra of native wheat starch and starch-oligosaccharides at different freezing-thaw cycles. [file mmc1.docx]
